# Virtual Reality Behavioral Activation for Adults With Major Depressive Disorder: Feasibility Randomized Controlled Trial

**DOI:** 10.2196/35526

**Published:** 2022-05-06

**Authors:** Margot Paul, Kim Bullock, Jeremy Bailenson

**Affiliations:** 1 PGSP-Stanford PsyD Consortium Palo Alto University Palo Alto, CA United States; 2 Department of Psychiatry & Behavioral Sciences Stanford School of Medicine Stanford, CA United States; 3 Department of Communication Stanford University Stanford, CA United States

**Keywords:** virtual reality, major depressive disorder, behavioral activation, depression

## Abstract

**Background:**

Major depressive disorder (MDD) is a global crisis with increasing incidence and prevalence. There are many established evidence-based psychotherapies (EBPs) for depression, but numerous barriers still exist; most notably, access and dissemination. Virtual reality (VR) may offer some solutions to existing constraints of EBPs for MDD.

**Objective:**

We aimed to examine the feasibility, acceptability, and tolerability of using VR as a method of delivering behavioral activation (BA) for adults diagnosed with MDD during a global pandemic and to explore for signs of clinical efficacy by comparing VR-enhanced BA (VR BA) to a standard BA treatment and a treatment as usual control group for individuals diagnosed with MDD.

**Methods:**

A feasibility trial using a 3-armed, unblinded, randomized controlled pilot design was conducted. The study took place remotely via Zoom telehealth visits between April 8, 2020, and January 15, 2021. This study used a 3-week, 4-session protocol in which VR BA participants used a VR headset to complete their BA homework. Feasibility was measured using dropout rates, serious adverse events, completion of homework, an adapted telepresence scale, the Simulator Sickness Questionnaire, the Brief Agitation Measure, and an adapted Technology Acceptance Model. Efficacy was assessed using the Patient Health Questionnaire–9.

**Results:**

Of the 35 participants assessed for eligibility, 13 (37%) were randomized into VR BA (n=5, 38%), traditional BA (n=4, 31%), or a treatment as usual control (n=4, 31%). The mean age of the 13 participants (5/13, 38% male; 7/13, 54% female; and 1/13, 8% nonbinary or third gender) was 35.4 (SD 12.3) years. This study demonstrated VR BA feasibility in participants with MDD through documented high levels of acceptability and tolerability while engaging in VR-induced pleasurable activities in conjunction with a brief BA protocol. No adverse events were reported. This study also illustrated that VR BA may have potential clinical utility for treating MDD, as the average VR BA participant’s clinical severity decreased by 5.67 points, signifying a clinically meaningful change in severity from a moderate to a mild level of depression as per the Patient Health Questionnaire–9 score.

**Conclusions:**

The findings of this study demonstrate that VR BA is safe and feasible to explore for the treatment of MDD. This study documented evidence that VR BA may be efficacious and justifies further examination in an adequately powered randomized controlled trial. This pilot study highlights the potential utility that VR technology may offer patients with MDD, especially those who have difficulty accessing real-world pleasant activities. In addition, for those having difficulty accessing care, VR BA could be adapted as a first step to help people improve their mood and increase their motivation while waiting to connect with a health care professional for other EBPs.

**Trial Registration:**

ClinicalTrials.gov NCT04268316; https://clinicaltrials.gov/ct2/show/NCT04268316

**International Registered Report Identifier (IRRID):**

RR2-10.2196/24331

## Introduction

### Background

Major depressive disorder (MDD) is a global crisis with increasing incidence and prevalence [1]. Depressive disorders are among the leading drivers of years lived with disability, and those who meet the criteria for MDD experience significant distress or impairment in areas of functioning [1,2].

Many evidence-based treatments have been identified for MDD [3]. Behavioral activation (BA) is considered one of the first-line treatments for MDD as the behavioral theory of depression states that a dearth of response-contingent positive reinforcement catalyzes symptoms of depression owing to less frequent engagement in pleasant activities or behavioral avoidance [3-5]. BA helps those who experience depression become less avoidant and more behaviorally activated by engaging in activities that are pleasurable or lead to a sense of accomplishment, which restores lost positive reinforcement and improves mood.

However, even in pre–COVID-19 times, only 56.8% of people diagnosed with MDD received some type of care to address their symptoms of depression over the course of 12 months [6]. For those who reach out for help, it is estimated that only 37.5% receive minimally adequate or evidence-based treatment [6]. Systemic barriers such as a lack of access to care and long wait times for appointments prevent individuals from engaging in mental health care [7]. Furthermore, there may be external obstacles that prevent those who experience MDD from engaging in BA, such as a lack of resources, financial constraints, physical limitations, and pandemic restrictions. The COVID-19 outbreak and subsequent widespread confinement to one’s home with *shelter-in-place* and community shutdown orders prevented individuals from partaking in enjoyable activities.

The use of technology as an adjunct to or a method of delivering mental health treatments is becoming increasingly popular, as technology can solve multiple barriers to care and grant increased access to evidence-based care when providers are unavailable [8]. One technology medium, virtual reality (VR), has been successfully used to help treat a variety of mental health conditions, with a study illustrating that VR video 360 was able to elicit similar emotional intensity and feelings of presence to real-life exposures [8,9]. Given the plethora of VR options readily available on the web for free and the cheaper headset selections, VR is now more publicly accessible than in previous years [10], and thus could help eliminate many of the aforementioned barriers to care.

Although using a VR headset presents minimal risk, studies have indicated that users may experience cybersickness, which may include symptoms such as headaches, nausea, dizziness, eye strain, reduced limb control, and reduced postural control [11-13]. However, there are ways to mitigate cybersickness, such as limiting prolonged continuous exposure to the virtual world [11].

### Objectives

VR-enhanced psychotherapy may enable increased access to BA by creating solutions to various barriers to engaging in pleasant activities, including pandemic restrictions and social isolation. The primary aim of this study was to examine whether using VR to engage in pleasurable activities within a BA protocol was a feasible, acceptable, and tolerable treatment. In addition, the study explored evidence of clinical efficacy in VR-enhanced BA for MDD compared with traditional BA and a treatment as usual (TAU) control. Finally, this study explored how mood was affected after partaking in a VR activity compared with engaging in an activity in real life.

## Methods

### Study Design

This was primarily a feasibility study conducted as a preliminary step in deciphering whether VR can be used as a method of delivering pleasurable or mastery activities during BA in a clinical sample of patients with MDD. This study was a 3-arm, nonblinded, between-participant, pilot randomized controlled trial (RCT) created to explore the initial feasibility and efficacy. This study aimed to recruit and enroll 30 participants and took place remotely via Zoom telehealth between April 8, 2020, and January 15, 2021.

### Participants

After gaining human-participant consideration and clearance from the Stanford Institutional Review Board (IRB-53483), participants were recruited from a study flyer posted at the Stanford School of Medicine, Department of Psychiatry and Behavioral Sciences, located in Palo Alto, California. The description of the study was also listed on the department’s currently recruiting studies website and on ClinicalTrials.gov. In addition, without solicitation, Curify, a health technology start-up based in San Francisco, placed study advertisements on Facebook without any formal agreement or payment from our research group. The inclusion criteria were as follows: aged ≥18 years; speaking English; and meeting the Diagnostic and Statistical Manual of Mental Disorders, Fifth Edition, criteria for MDD. The exclusion criteria were as follows: a substance use disorder in the past year, diagnosis of any psychotic or bipolar I disorder, seizure in the last 6 months or untreated epilepsy, current suicidal urges or intent, or current nonsuicidal self-injury or parasuicidal behavior.

### Procedures

#### Overview

The initial screening procedure consisted of 2 steps: an initial phone screen and a face-to-face Zoom intake session. During the phone screen, callers were briefly assessed for initial eligibility and provided with the opportunity to ask questions about the study. Initial eligibility was determined by a Patient Health Questionnaire–8 score of ≥10 [14] as well as a brief questionnaire. If eligible and still interested in participation, a formal initial intake was scheduled via Zoom, and the participants were securely emailed the consent form for review before the meeting. After asking any questions and securely emailing the signed consent form back to the protocol director, the intake session occurred. During the Zoom intake session, the participants were asked to verbally complete a demographic questionnaire while the protocol director shared her screen via Zoom. The participants were subsequently administered the Mini-International Neuropsychiatric Interview by the protocol director. The participants were then informed of their eligibility and, if eligible, scheduled for their first session via Zoom. See the previously published case report [15] for further details.

#### Randomization

The participants were randomly assigned to receive one of the 3 study arms in a single-blind fashion by using permuted blocks of 6 in sealed envelopes. A target sample size of 30 patients was selected in keeping with the higher end of the range of sample sizes used for such feasibility studies.

#### Intervention

At the beginning of each session, all participants were verbally administered the Patient Health Questionnaire–9 (PHQ-9). The protocol director shared her screen over Zoom with the participants in the VR BA and traditional BA arms while collecting this measure. The participants in the TAU arm were only read the questions over the phone. If item 9 was endorsed, a risk assessment was conducted in real time, and proper measures were taken in accordance with risk.

#### Intervention: Treatment as Usual Arm

After the 4 meetings were completed, these participants were given the option to meet once with the protocol director via Zoom for 50 minutes so that the protocol director could explain the theory behind BA, provide psychoeducation around pleasurable and mastery activities, and explain how the participants could incorporate BA into their lives. The participants also had the option to receive a Google Cardboard as an incentive to remain in the TAU control group. The protocol director explained how to use the Google Cardboard as a potential method of engaging in pleasurable activities. Only the data accrued during the 4 meetings were used in the study.

#### Intervention: VR BA and Traditional BA Arms

These participants met with the protocol director 4 times, once per week for 3 weeks, over Zoom for 50 minutes to receive BA therapy. The VR participants were shipped a VR headset before the first session. The headset was supplied by Limbix, now partnered with BehaVR. This headset had a 5.5-inch screen size with a resolution of 2560 x 1440 pixels, a screen aspect ratio of 16:9, a field of view of 92 degrees, 3 *df*, and a refresh rate of 70 Hz. See the previously published case report for further description of the VR device [15]. Both arms followed the protocol for brief BA based on the guidance of published literature [16,17]. The treatment incorporated 4 components: establishing the therapeutic relationship, developing goals for treatment, conducting a functional analysis, and treatment review with relapse prevention [17].

The first session focused on establishing rapport, identifying activities that the participants valued or had felt a sense of mastery or pleasure from in the past, introducing the mood-activity log, and setting activity goals [16]. The traditional BA participants were provided with an in-person activity list and required to schedule *real life* activities, whereas the participants in the VR BA arm were provided with VR activity options and required to choose VR activities for the week. These VR activities consisted of 360-degree videos that did not entail the participants’ active involvement but were simulations of activities that were passively watched, other than allowing the users to change their visual perspectives with head movements. The VR BA participants were also asked to complete a post-VR questionnaire assessing spatial presence, simulator sickness, agitation, and acceptability after each VR activity.

During sessions 2 and 3, the protocol director reviewed the mood-activity log (session 2) and activity schedule (session 3) with the participants and checked in regarding goal attainment to reinforce homework completion [18]. The participants in the VR BA and traditional BA arms were asked to rate their mood on a scale of 1 to 10 (1=worst they ever felt and 10=best they ever felt) before and after their chosen activity. Barriers to completion of activities and problem-solving strategies were again discussed, and new activity goals were introduced and scheduled. During session 4, the treatment and skills were reviewed, and feedback was provided by participants. For further details, see the previously published case report [15].

### Outcomes

#### Feasibility

Feasibility was assessed using dropout rates, serious adverse events reported, completion of homework, and level of presence experienced in the headset. Sense of presence is a psychological construct and is used as a measure of the ecological validity of VR devices. Sense of presence is defined as a “sense of being there” or a “feeling of being in a world that exists outside of the self” [19]. This presence questionnaire is a validated measure that is correlated with procedural learning enhancement. Dropout rates were assessed using the number of individuals who did not complete the full 4-session protocol after randomization. Serious adverse events were gathered from qualitative interviews and notes. Completion of homework in the VR BA arm was determined by the number of times the headset was used and the number of times the post-VR questionnaire was completed. The number of times the headset was used was obtained from the data collected from the headset after participant termination or completion of the study. The number of post-VR questionnaires completed was calculated from the number of post-VR questionnaires that each participant emailed to the protocol director. The participants were asked to complete ≥4 VR activities per week and a post-VR questionnaire for each VR activity completed. Completion of homework in the traditional BA arm was defined as completing the mood-activity log after session 1 and completing ≥4 activities in real life each week after sessions 2 and 3. These data were collected via participant reports and the completed mood-activity log and activity tracking forms that were emailed to the protocol director. See the previously published case report [15] for information on the presence scale.

#### Mood

See the previously published case report [15] for information about the PHQ-9. An exploratory measure of mood was obtained before and after participating in the BA activity of choice by answering the following question—*How would you rate your current mood*—ranging from 1 (worst ever felt) to 10 (best ever felt). This was adapted from the single-item self-rating scale of happiness, which has good reliability (0.86) and construct validity (Cronbach *α*=.55-.94) [20].

See the previously published case report [15] for information on the following outcome measures: demographics, the Mini-International Neuropsychiatric Interview, acceptability, and tolerability.

### Statistical Methods

A power analysis was deemed unnecessary given that the primary purpose of the study was to assess the feasibility of using VR to engage in pleasurable or mastery activities as an adjunct to a brief BA protocol. The feasibility, or the degree to which VR could successfully be integrated into the brief BA protocol, was measured by commenting on qualitative barriers to use observed. Barriers were assessed by rates of dropout, adverse events, and the number of times the headset was used. The level of presence was obtained via participant reports from a Likert scale of 0 (not at all) to 4 (very strongly) for each question; with 3 questions, there was a possibility of yielding a score between 0 and 12. The average total presence for each participant, intention-to-treat (ITT) participant, and protocol completer was then calculated. The average presence experienced was also calculated as a percentage by dividing the average score by 12 (the maximum score).

Acceptability of the VR BA treatment was measured via participant reports using the Technology Acceptance Model, with the agreeance choice on a Likert scale ranging from 0 (strongly disagree) to 4 (strongly agree). The number of questions in each category determined the outcome range (either 0-12 for 3 questions or 0-16 for 4 questions). Each participant’s scores were then averaged along with the average ITT participants’ and protocol completers’ scores. The average percentage of acceptance was also calculated by dividing the average score by the maximum score in the outcome range. To determine the degree of acceptance as labeled on the scale, the average score was then scaled back depending on the number of questions. For example, the *Perceived Usefulness* category included 3 questions, yielding a potential range of 0 to 12, so an average score of 10 would be divided by 3 to assess the degree of acceptance (in this case, it would yield a score of 3.33, which would correlate to *agree* on the Likert scale).

Physical tolerability of the VR headset was assessed via participant reports using the Simulator Sickness Questionnaire, and the emotional tolerability of the VR headset was assessed via participant reports using the Brief Agitation Measure. Physical tolerability was broken into each item and ranged from 0 (no more than usual) to 3 (severely more than usual) for each item. Each participant’s scores were averaged along with the average ITT participants’ and protocol completers’ scores. The total percentage tolerability rating for a given activity was calculated by dividing a participant’s score by 48, as there were 16 items, yielding a potential range of 0 to 48. The percentage of intolerability for each symptom category was similarly calculated by dividing the average score by the maximum potential score of 3. The average scores for physical tolerability were summed for each participant along with the average emotional tolerability scores of each participant. Emotional tolerability was scored from 1 (strongly disagree) to 7 (strongly agree) per question; with 3 questions, there was a possibility of yielding a score between 3 and 21. These scores were rescaled to a range of 0 to 18 by subtracting 3 from all scores. The percentage of physical and emotional intolerability was calculated by dividing the average scores by the highest potential score (48 for physical tolerability and 18 for emotional tolerability).

To assess the clinical efficacy of the VR BA treatment compared with the traditional BA and TAU control groups, the participants’ depression scores were measured using the PHQ-9 at 4 time points. Owing to the small sample size, statistical analyses were not used; rather, each group’s mean score was graphically represented across time.

To explore whether engaging in an activity in VR increased mood more than engaging in an activity in real life, the participants were asked to rate their mood on a scale of 1 to 10 (1=worst they ever felt and 10=best they ever felt) before and after their chosen activity. The differences in mood before and after each VR activity were cumulatively added across each participant and then divided by the number of activities completed to find the mean. The same was done for the activities completed after sessions 2 and 3 (when the participants were asked to track their pre- and postactivity moods) for the traditional BA group. In addition, the reported pre- to postactivity mood changes of the participants in the VR BA and traditional BA groups were tallied and graphically represented.

### Ethics Approval

This study was approved by Stanford University’s IRB (protocol #53483) and registered on ClinicalTrials.gov (ID #NCT04268316). A CONSORT (Consolidated Standards of Reporting Trials) checklist is also included in Multimedia Appendix 1.

## Results

### Participant Demographics

**Figure 1:**
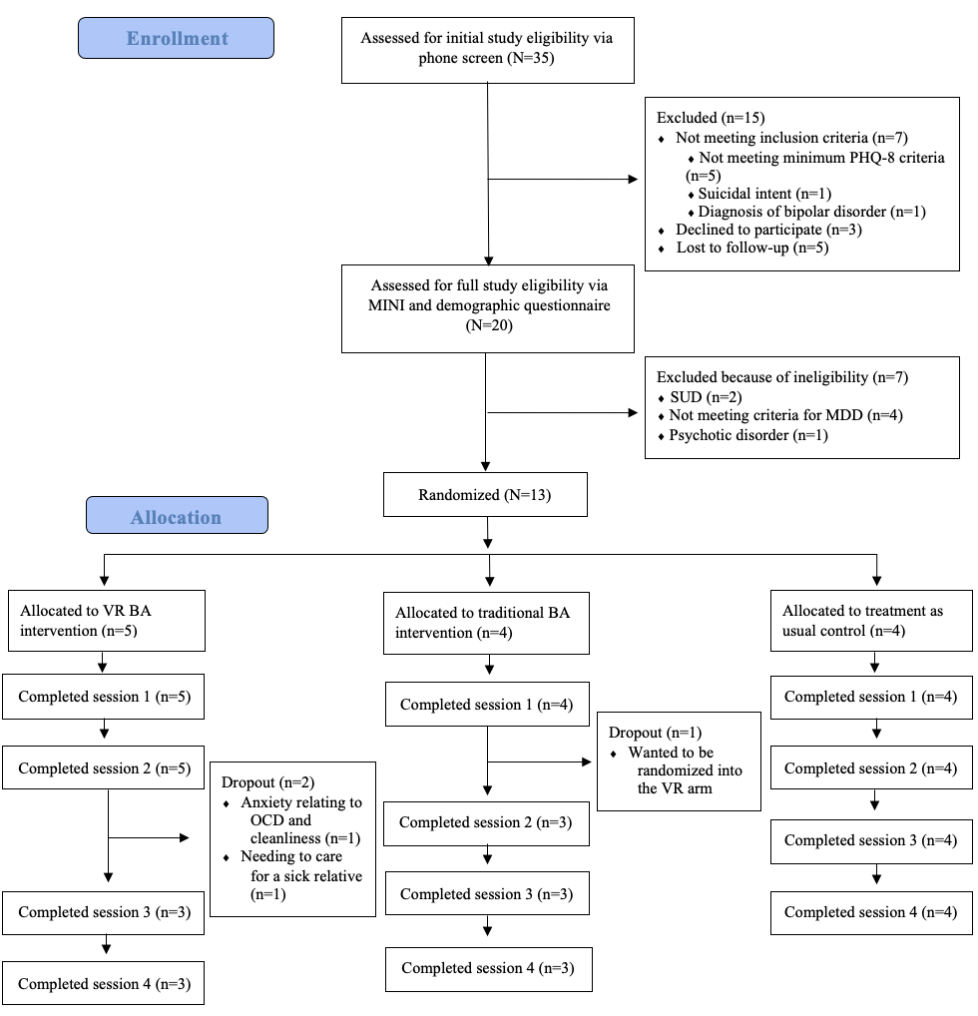
CONSORT (Consolidated Standards of Reporting Trials) diagram. BA: behavioral activation; MDD: major depressive disorder; MINI: Mini-International Neuropsychiatric Interview; OCD: obsessive-compulsive disorder; PHQ-8: Patient Health Questionnaire–8; SUD: substance use disorder; VR: virtual reality.

The sample consisted of 13 adults (mean age 35.4, SD 12.3 years; 5/13, 38% male; 7/13, 54% female; and 1/13, 8% nonbinary or third gender), with 10 (77%) adults (mean age 34.6, SD 11.50 years; 5/10, 50% male; 4/10, 40% female; and 1/10, 10% nonbinary or third gender) completing the full protocol. See Figure 1 for the CONSORT diagram and Table 1 for more participant demographic information.

**Table 1:** Participant demographics (N=13).

Characteristics				VR^a^ BA^b^ (n=5), n (%)		Traditional BA (n=4), n (%)		TAU^c^ control (n=4), n (%)		Total, n (%)
**Gender**										
	Male		1 (20)		3 (75)		1 (25)		5 (38)	
	Female		4 (80)		1 (25)		2 (50)		7 (54)	
	Nonbinary or third gender		0 (0)		0 (0)		1 (25)		1 (8)	
**Age (years)**										
	20 to 25		1 (20)		1 (25)		2 (50)		4 (31)	
	26 to 30		2 (40)		0 (0)		0 (0)		2 (15)	
	31 to 40		0 (0)		0 (0)		2 (50)		2 (15)	
	41 to 45		2 (40)		1 (25)		0 (0)		3 (23)	
	51 to 55		0 (0)		1 (25)		0 (0)		1 (8)	
	56 to 60		0 (0)		1 (25)		0 (0)		1 (8)	
**Race or ethnicity**										
	Non-Hispanic White		3 (60)		3 (75)		1 (25)		7 (54)	
	Chinese		1 (20)		1 (25)		0 (0)		2 (15)	
	Indian		1 (20)		0 (0)		0 (0)		1 (8)	
	African American		0 (0)		0 (0)		1 (25)		1 (8)	
	Mexican		0 (0)		0 (0)		1 (25)		1 (8)	
	Hispanic or Latino		0 (0)		0 (0)		1 (25)		1 (8)	
**Past mental health treatment**										
	**Yes**		5 (100)		4 (100)		4 (100)		13 (100)	
		Psychotherapy only	0 (0)		0 (0)		1 (25)		1 (8)	
		Psychotropic medications only	0 (0)		0 (0)		0 (0)		0 (0)	
		Psychotherapy and medications	5 (100)		4 (100)		3 (75)		12 (92)	
	No		0 (0)		0 (0)		0 (0)		0 (0)	
**Current mental health treatment**										
	**Yes**		4 (80)		4 (100)		3 (75)		11 (85)	
		Psychotherapy only	0 (0)		1 (25)		1 (25)		2 (15)	
		Psychotropic medications only	2 (40)		0 (0)		0 (0)		2 (15)	
		Psychotherapy and medications	2 (40)		3 (75)		2 (50)		7 (54)	
	No		1 (20)		0 (0)		1 (25)		2 (15)	
**Previous experience using VR**										
	0 times		2 (40)		1 (25)		2 (50)		5 (38)	
	1 to 4 times		3 (60)		2 (50)		2 (50)		7 (54)	
	5 to 9 times		0 (0)		0 (0)		0 (0)		0 (0)	
	≥10 times		0 (0)		1 (25)		0 (0)		1 (8)	
**Purpose of past VR use**										
	Gaming		2 (40)		2 (50)		2 (50)		6 (46)	
	Treatment		0 (0)		1 (25)		0 (0)		1 (8)	
	Research		0 (0)		0 (0)		1 (25)		1 (8)	
	Other (conferences)		1 (20)		0 (0)		0 (0)		1 (8)	

^a^VR: virtual reality.

^b^BA: behavioral activation.

^c^TAU: treatment as usual.

#### VR BA Feasibility

The completion rate was 60% (3/5) in the VR BA arm, 75% (3/4) in the traditional BA arm, and 100% (4/4) in the TAU control arm. No participants reported any serious adverse events. The participants in the VR BA arm used the headset, on average, more than required (Table 2). Of the 5 participants, 2 (40%)—participant 4 and participant 28—noted that they kept the VR headset nearby so that they could more readily access it and remember to use it. However, only 20% (1/5) of the participants completed a post-VR questionnaire after each VR activity, with the other participants completing less than required. Participant 24 specifically expressed difficulty disentangling headset use with completing the post-VR questionnaires, which she found stressful and tedious to complete.

**Table 2:** Virtual reality behavioral activation feasibility.

	Dropout (yes or no)	Adverse events, N	Completed mood activity log (yes or no)	Times headset was used between session 1 and session 4^a^, N	Completed homework worksheets^a^, N	Level of presence experienced in headset^b^ (0-12; 3 items), mean (SD)
Participant 4	No	0	Yes	21	15	9.53 (1.96)
Participant 12	No	0	Yes	11	11	2.82 (2.99)
Participant 24	Yes	0	Yes	11	5	6.40 (1.82)
Participant 28	No	0	Yes	33	9	9.56 (3.88)
Participant 30	Yes	0	No	5	1	7.00 (N/A)
Completer average	N/A^c^	0	N/A	21.67	11.67	7.30 (3.88)
ITT^d^ average	N/A	0	N/A	16.20	8.20	7.06 (2.77)

^a^Minimum required headset use and completed homework worksheets was 12 each.

^b^Level of presence contained 3 items with a range of 0 (not at all) to 4 (very strongly) for each item. Higher numbers indicate greater presence.

^c^N/A: not applicable.

^d^ITT: intention-to-treat.

The average total presence rating of the ITT VR BA participants was 59% (7.06/12), whereas the average rating of all the VR BA protocol completers was 61% (7.30/12; Table 2). Participant 24, who reported an average presence rating of 53% (6.40/12), noted that she had difficulty using the head-mounted display (HMD) with her glasses as it led to smudging. Participant 12, who reported a comparatively lower average presence rating of 24% (2.82/12), stated that she “wanted more control of when to stop in the video and look around” and wanted the ability to interact in the virtual environment. She also remarked that the image quality of the videos was not as good as that of real-life imagery. Participant 12 further noted that there was a problem in the lower left visual field of her VR headset, greatly impairing her sense of presence.

#### VR BA Acceptability

Overall, the participants who completed the protocol “agreed” that the VR treatment was acceptable, with an average rating of 87% (45.32/52) acceptability, and all VR BA participants (5/5, 100%) verbally provided positive endorsements for using the headset (Table 3).

**Table 3:** Virtual reality behavioral activation acceptability.

	Perceived usefulness^a^ (0-12; 3 items), mean (SD)	Perceived ease of use^a^ (0-12; 3 items), mean (SD)	Attitudes toward use^b^ (0-16; 4 items), mean (SD)	Intention to use technology^a^ (0-12; 3 items), mean (SD)
Participant 4	11.00 (0)	12.00 (0)	16.00 (0)	12.00 (0)
Participant 12	7.00 (1.41)	8.90 (0.32)	8.10 (3.63)	9.30 (0.95)
Participant 24	8.80 (1.48)	10.60 (0.89)	11.20 (2.95)	6.40 (1.52)
Participant 28	11.67 (1.00)	12.00 (0)	16.00 (0)	12.00 (0)
Participant 30	10.00 (N/A^c^)	10.00 (N/A)	11.00 (N/A)	8.00 (N/A)
Completer average	9.89 (2.52)	10.97 (1.79)	13.37 (4.56)	11.10 (1.56)
ITT^d^ average	9.69 (1.85)	10.70 (1.33)	12.46 (3.46)	9.54 (2.47)

^a^Domains comprising the technology acceptance model (higher numbers indicate greater acceptability). Perceived usefulness, perceived ease of use, and intention to use technology contained 3 items with a range of 0 (strongly disagree) to 4 (strongly agree) for each item.

^b^Attitudes toward use contained 4 items with a range of 0 (strongly disagree) to 4 (strongly agree) for each item.

^c^N/A: not applicable

^d^ITT: intention-to-treat.

#### VR BA Tolerability

The average overall physical tolerability of those who completed the full protocol and the ITT participants was 92% (44.23/48) and 94% (45.06/48), respectively (Table 4). *Nausea* was the most endorsed symptom of physical intolerability (Table 5). *Burping* was the least endorsed symptom of physical intolerability, with no participants endorsing it after any activity. Participant 30 stated that she becomes seasick/carsick easily and found some of the VR activities nauseating. Participant 12 informed that she also becomes carsick easily and not being in control of the image’s movement made her feel sick until the headset was removed, with the longest lingering symptom dissipating 30 minutes after headset removal. The average overall emotional tolerability of those who completed the full protocol and the ITT participants was 90% (16.21/18) and 94% (16.93/18), respectively (Table 4).

**Table 4:** Overall tolerability.

	Physical tolerability^a^ (0-48; 16 items), total mean^b^ (SD)	Emotional tolerability^c^ (0-18; 3 items), total mean^d^ (SD)
Participant 4	1.73 (0.14)	0.00 (0)
Participant 12	8.73 (0.23)	5.36 (0.14)
Participant 24	0.40 (0.07)	0.00 (0)
Participant 28	0.78 (0.10)	0.00 (0)
Participant 30	3.00 (N/A^e^)	0.00 (0)
Completer average	3.75 (4.34)	1.79 (3.10)
ITT^f^ average	2.93 (3.39)	1.07 (2.40)

^a^Physical tolerability determined using the Simulator Sickness Questionnaire. Possible responses for the 16 items ranged from 0 (no more than usual) to 3 (severely more than usual). Lower numbers indicate greater tolerability.

^b^The mean scores for physical tolerability were summed for each participant.

^c^Emotional tolerability determined using the Brief Agitation Measure. Possible responses for the 3 items ranged from 0 (strongly disagree) to 6 (strongly agree). Lower numbers indicate greater tolerability.

^d^The mean scores for emotional tolerability were summed for each participant.

^e^N/A: not applicable.

^f^ITT: intention-to-treat.

**Table 5:** Physical tolerability.

	Participant 4, mean (SD)	Participant 12, mean (SD)	Participant 24, mean (SD)	Participant 28, mean (SD)	Participant 30, mean (SD)	Completer average, mean (SD)	ITT^a^ average, mean (SD)
Nausea^b^ (0-3)	0.33 (0.62)	0.91 (1.22)	0.20 (0.40)	0.22 (0.44)	1 (N/A^c^)	0.49 (0.37)	0.53 (0.39)
General discomfort^b^ (0-3)	0.20 (0.56)	0.91 (1.22)	0 (0)	0 (0)	0 (N/A)	0.37 (0.48)	0.22 (0.39)
Stomach awareness^b^ (0-3)	0.27 (0.70)	0.73 (1.27)	0 (0)	0.33 (0.71)	0 (N/A)	0.44 (0.25)	0.27 (0.30)
Sweating^b^ (0-3)	0.27 (0.70)	0.55 (1.21)	0 (0)	0.11 (0.33)	0 (N/A)	0.31 (0.22)	0.19 (0.23)
Increased salivation^b^ (0-3)	0.13 (0.35)	0.55 (1.21)	0 (0)	0 (0)	0 (N/A)	0.23 (0.28)	0.14 (0.24)
Vertigo^b^ (0-3)	0.33 (0.90)	0.55 (1.21)	0 (0)	0.11 (0.33)	0 (N/A)	0.33 (0.22)	0.20 (0.24)
Burping^b^ (0-3)	0 (0)	0 (0)	0 (0)	0 (0)	0 (N/A)	0 (0)	0 (0)
Difficulty concentrating^b^ (0-3)	0 (0)	0.45 (0.82)	0 (0)	0 (0)	1 (N/A)	0.15 (0.26)	0.29 (0.44)
Difficulty focusing^b^ (0-3)	0 (0)	0.45 (0.82)	0 (0)	0 (0)	0 (N/A)	0.15 (0.26)	0.09 (0.20)
Eye strain^b^ (0-3)	0 (0)	0.55 (1.21)	0.20 (0.40)	0 (0)	0 (N/A)	0.18 (0.31)	0.15 (0.24)
Fatigue^b^ (0-3)	0 (0)	0.18 (0.40)	0 (0)	0 (0)	0 (N/A)	0.06 (0.10)	0.04 (0.08)
Headache^b^ (0-3)	0 (0)	0.64 (1.21)	0 (0)	0 (0)	1 (N/A)	0.21 (0.37)	0.33 (0.47)
Blurred vision^b^ (0-3)	0 (0)	0.36 (0.81)	0 (0)	0 (0)	0 (N/A)	0.12 (0.21)	0.07 (0.16)
Dizziness (eyes open^b^; 0-3)	0.20 (0.56)	0.64 (1.21)	0 (0)	0 (0)	0 (N/A)	0.28 (0.33)	0.17 (0.28)
Dizziness (eyes closed^b^; 0-3)	0 (0)	0.64 (1.21)	0 (0)	0 (0)	0 (N/A)	0.21 (0.37)	0.13 (0.28)
Fullness of head^b^ (0-3)	0 (0)	0.64 (1.21)	0 (0)	0 (0)	0 (N/A)	0.21 (0.37)	0.13 (0.28)

^a^ITT: intention-to-treat.

^b^Symptoms included in the Simulator Sickness Questionnaire. Each symptom had a range of 0 (no more than usual) to 3 (severely more than usual). Lower numbers indicate greater tolerability.

^c^N/A: not applicable.

#### Clinical Efficacy

**Figure 2:**
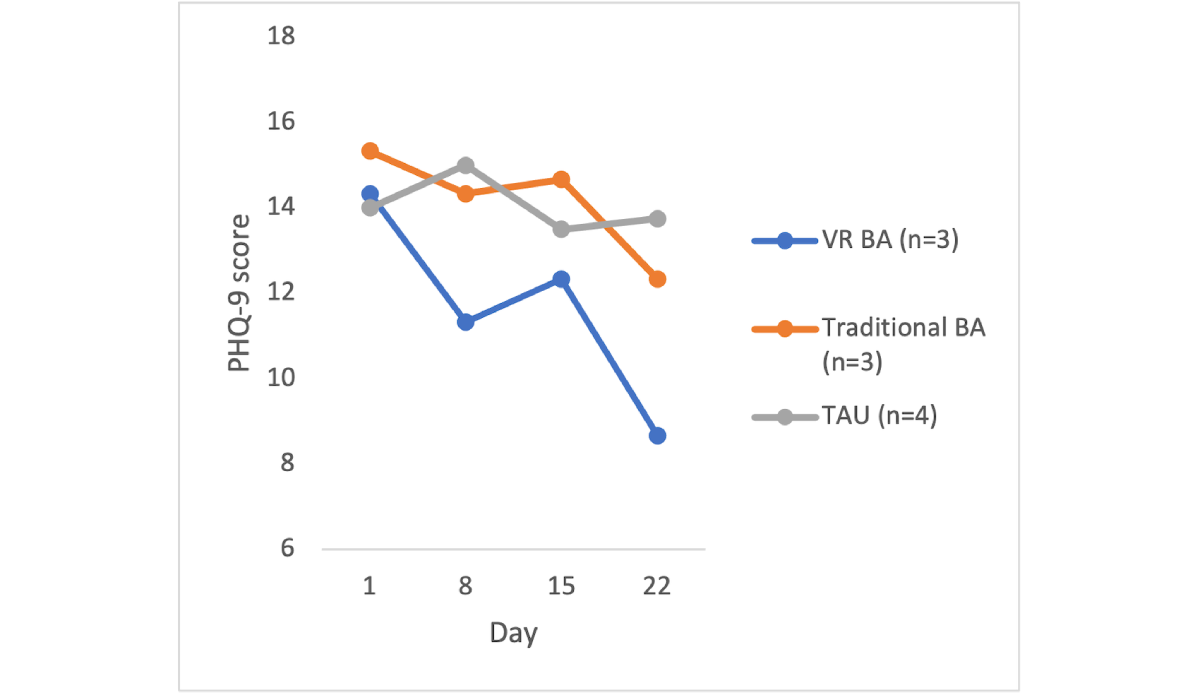
Average Patient Health Questionnaire–9 (PHQ-9) score across time. BA: behavioral activation; TAU: treatment as usual; VR: virtual reality.

Owing to a lower than anticipated sample size, there was not enough power to conduct statistical analyses, and a graphical representation was used. Figure 2 shows the PHQ-9 scores of the participants who completed the full 4-session protocol. Overall, the mean PHQ-9 scores of the VR BA group decreased by 5.67, changing the average diagnostic severity category rating from moderate depression (14.33) to mild depression (8.67; Figure 2), a clinically significant change (>5) [14]. The mean PHQ-9 scores of the traditional BA group decreased by 3, changing the average severity from moderately severe depression (15.33) to moderate depression (12.33). The mean PHQ-9 scores of the TAU control group decreased by 0.25, which did not change the average diagnosis severity level (moderate depression).

#### Pre- to Postactivity Mood Scores

**Figure 3:**
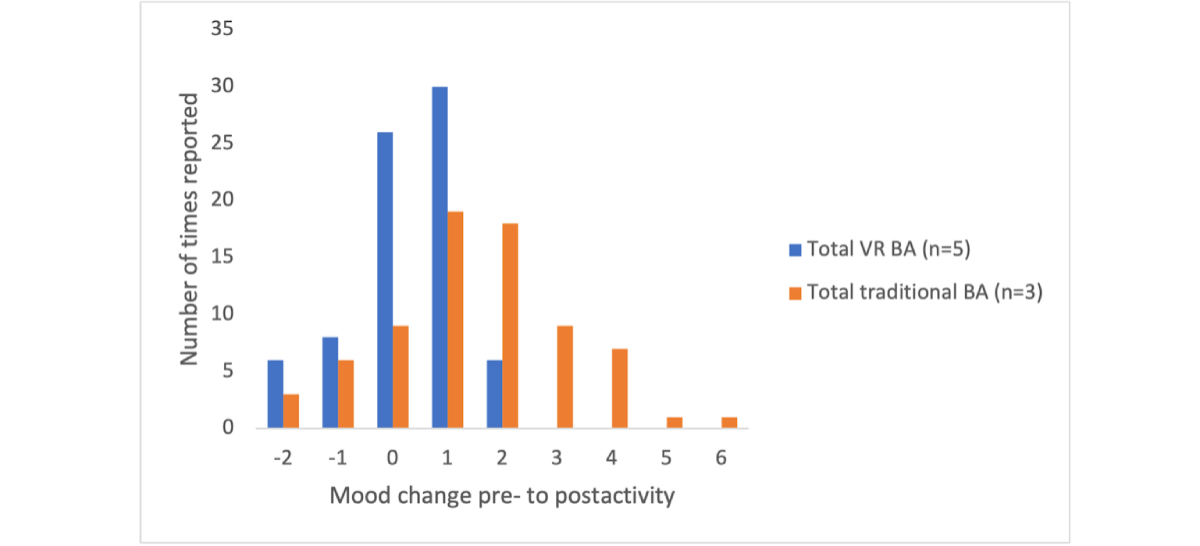
Participant pre- to postactivity mood changes. BA: behavioral activation; VR: virtual reality.

Descriptive statistics of the pre- to postactivity mood scores between the VR BA and traditional BA groups are presented in Table 6. The mean change in mood reported by the participants who completed the VR BA protocol was 0.18, whereas the mean change in mood reported by the participants who completed the traditional BA protocol was 1.48 (Table 6). The mode-reported mood change was 1 among both the VR BA and traditional BA participants (Figure 3). The lowest reported mood change among both the VR BA and traditional BA participants was −2, whereas the highest reported mood change was 2 among the VR BA participants and 6 among the traditional BA participants.

**Table 6:** Average change in mood scores pre- to postactivity completion.

VR^a^ BA^b^ participant	Change in mood after VR activity^c^, mean (SD)	Traditional BA participant	Change in mood after real-life activity^d^, mean (SD)
Participant 4	0.71 (0.85)	Participant 14	1.58 (1.89)
Participant 12	−0.36 (1.21)	Participant 21	0.65 (1.46)
Participant 24	0.36 (1.12)	Participant 22	2.20 (1.06)
Participant 28	0.18 (0.86)	Participant 23	N/A^e^
Participant 30	0.40 (1.52)	N/A	N/A
Completer average	0.18 (0.54)	Completer average	1.48 (0.78)
ITT^f^ average	0.26 (0.40)	Total average	N/A

^a^VR: virtual reality.

^b^BA: behavioral activation.

^c^The observed minimum change in the VR BA group was −2, and the observed maximum change was 2.

^d^The observed minimum change in the traditional BA group was −1, and the observed maximum change was 6.

^e^N/A: not applicable.

^f^ITT: intention-to-treat.

## Discussion

### Principal Findings

The results of this study illustrate that VR is a feasible, acceptable, and tolerable method of engaging in pleasurable activities in conjunction with a BA intervention for MDD. The attrition rate of 23% (3/13) of the participants in this study is comparable with other VR studies [21,22], lower than that of many RCTs of internet-based interventions for depression [23], and lower than that of a small-sample pilot RCT exploring exercise as a treatment for depression [24]. None of the participants in the VR BA treatment arm dropped out of the study because of adverse events, and no adverse events were reported throughout the duration of the study.

Despite the COVID-19 pandemic, on average, the participants in the VR BA and traditional BA arms complied with the homework assignment of completing ≥4 activities each week. However, only 20% (1/5) of the participants in the VR BA arm completed a post-VR questionnaire for every VR activity completed. This lack of full questionnaire completion could be due to the repetitive nature of the questions, as several participants noted survey fatigue. This may also be the reason why the participants in the traditional BA arm reportedly engaged in more activities than the participants in the VR BA arm as those in the traditional BA arm did not need to complete a post-VR questionnaire equivalent after each activity; rather, they were simply required to document their pre- and postactivity mood.

Another potential reason for the shortage of questionnaire completion may have been that the study was conducted via telehealth. It is possible that providing hard copies of the post-VR questionnaire in person at the end of each session and collecting these copies at the beginning of the following session may have yielded an increase in post-VR questionnaire completion [18].

Although the VR presence ratings were lower than expected, they were comparable with the presence ratings in other VR studies [21]. In general, presence may not have been higher for a few reasons. First, the Limbix headset created a subtle effect that one is looking at the image through a screen owing to the simple device technology. Second, in using a 360-degree video, to give the illusion of movement, the image moves while the participant remains still rather than the participant being able to walk around the virtual environment. Participant 12 even noted that she wished she could interact more with the environment and wanted the autonomy to decide when to stop and look around. She stated that she would have preferred a digitally rendered environment that was interactive over a more realistic environment that did not have interactive capabilities, which aligns with research illustrating that interactivity is more important than realism for yielding a greater sense of presence [25]. Furthermore, some of the activities involved sounds that were not natural to or consistent with the environment, such as a voice-over description of the scene or gentle music playing in the background. Although this HMD was chosen for its simplicity of use and portability, it is possible that, with a more advanced device or one with greater interactivity, the presence ratings would be higher [25]. However, the presence ratings were not correlated with the participants’ pre- to post-VR mood ratings in that a lower presence rating could yield a greater increase in mood than a higher presence rating and vice versa, a finding consistent with the literature given the nature of the emotion [26].

Despite the presence ratings potentially being affected by the device simplicity, on average, protocol completers *strongly agreed* that the VR device was easy to use and *agreed* that the VR BA protocol was useful. These findings are consistent with the literature stating that the simpler and easier-to-use the VR device is, the more useful it will be [27].

The participants rated the protocol as largely physically tolerable, with an average tolerability rating of 92% (44.23/48) among the protocol completers and 94% (45.06/48) among the ITT participants, and no participants dropped out because of adverse effects. Consequently, using VR to decrease symptoms of depression may be more tolerable than taking antidepressant medications, with participants in antidepressant trials dropping out because of side effects [28]. However, larger-scale VR trials must be completed to better assert this claim.

Participant 12, who endorsed the lowest physical tolerability, specifically attributed her cybersickness to her not being in control of the image’s movement. The fact that most of the cybersickness symptoms and the strongest reported intensity of symptoms occurred during the adrenaline activities may indicate that it was due to the mismatch between the participants’ vestibular and visual cues as the movement of the image during adrenaline activities happens more quickly than during the other activities, such as watching a sunset or observing nature [11,13]. Despite being the only participant to report symptoms of agitation, participant 12 did not drop out of the study, and the symptoms did not correlate with her reported mood changes pre- to post-VR activity. Participant 12’s report of this emotional intolerability while using the headset may be due to frustration around the aforementioned problem with the visual field of the headset, and her subsequent endorsement of sadness may be due to wishing she was in the physical space of the activity.

Although the sample size was not large enough to statistically comment on whether the fidelity and efficacy of BA withstands the modification of BA to a VR format, the initial signal supports the possibility that it is not inferior. In this sample, VR BA participants experienced a greater decrease in PHQ-9 scores than those who completed the traditional BA or the TAU controls. The overall clinical severity (>5) [14] decrease in depressive symptoms for those in the VR BA arm illustrated that, despite the restrictions in place because of the COVID-19 pandemic, the participants were able to meaningfully clinically improve using VR BA.

The mean scores in the traditional BA group also decreased, with the average severity changing from moderately severe (15.33) to moderate depression (12.33). This aligns with the literature illustrating that a brief BA protocol can decrease symptoms of MDD. Although the change was not considered *clinically* significant as it did not meet the threshold of at least a 5-point decrease [14,16,29] per PHQ-9 criteria, this decrease in symptoms, which shifted the diagnostic categories, is a good indicator of the fidelity of the traditional BA group protocol.

This discrepancy in PHQ-9 scores between traditional BA and VR BA may have occurred because of the small sample size, and thus may not be significant. These results may also be due to the fact that the VR BA participants could have been more excited than the traditional BA participants when completing their activities—the VR BA participants noted that the novelty of using the HMD was “exciting,” whereas no such equivalent was noted among the traditional BA participants. Furthermore, the BA participants did not have the opportunity to engage in VR activities, whereas the VR BA participants were not discouraged from partaking in real-life activities. Notably, 40% (2/5) of the participants informed the protocol director that they were more motivated to partake in real-life activities after using the headset. Therefore, it is possible that the VR BA participants increased their activities in real life in addition to using the VR headset. This could explain the fact that, although the VR BA group endorsed less of an average change in mood pre- to postactivity measurement compared with the traditional BA group, they still experienced a numerically greater decrease in depression symptoms.

If using VR can improve mood or at least provide enough of a boost in mood to increase one’s motivation to engage in other pleasurable or mastery activities, it could greatly decrease the burden that depression has on individuals and society. This use could also provide some symptom relief for individuals waiting to see a mental health care provider. Furthermore, once an individual is in therapy, the use of VR could provide a sense of novelty, which may encourage individuals struggling with symptoms of depression to engage in the intervention [30]. Thus, providers could consider incorporating VR as a first step in a hierarchy of activity scheduling to incrementally increase their clients’ behavioral motivation. Scheduling activities was not an easy feat during the COVID-19 global pandemic, and using VR as a means of engaging in activities that otherwise could not be explored provided excitement and “escape” for the participants and could continue to do so if preventative barriers occur in the future. Finally, although previous studies have illustrated that BA has higher rates of retention than antidepressant medications among patients who were more severely depressed, this study further illustrated that VR may be more tolerable than antidepressant medications [4,28]. This finding suggests that partaking in a VR BA protocol could be a potential treatment alternative for those who have failed psychiatric medications owing to the side effects.

Going forward, it is necessary to replicate this study with a larger sample size both to confirm the findings and to statistically assess the efficacy and effectiveness compared with traditional BA. In addition, although this study used a Limbix HMD with videos already preloaded onto the headset for ease of use and controllability, it would be interesting to conduct a similar study with some of the more easily accessible, interactive, and immersive content with less expensive headset options. Headsets such as Google Cardboard could provide greater accessibility and content variety for the general population to engage with VR and potentially experience these positive changes.

Furthermore, given the feedback from some participants that they would have preferred more interaction within the VR landscape rather than passively watching the environment around them, research comparing the use of different headsets on feasibility, acceptability, tolerability, and efficacy is needed. This would provide additional data for individuals who have the option of obtaining different headsets and allow them to choose the option to best fulfill their wants and needs. Moreover, future research could incorporate HMDs with options to interact with other users and assess whether the social engagement component is correlated with an increase in mood. This methodology would provide a more realistic opportunity for pleasurable activities for some individuals whose values align with being social, as well as greater social accessibility for people who encounter barriers to engaging in social interaction, such as pandemic restrictions. In addition, a more advanced headset could potentially provide more activity choices, enabling individuals to engage in activities that align with their values and potentially increasing their mood.

Finally, although this study was open to adults aged >18 years, the age range of the VR BA participants was 20 to 41 years. Given that older adults experience an increase in prevalence of MDD after the age of 85 years, especially when residing in a hospital or long-term care facility setting [31], and older adults in these settings often have barriers that prevent them from becoming behaviorally activated in real life, it is important to conduct a VR BA study similar to this one with older adults. If older adults were able to experience an increase in positive mood after using VR in a similar vein to the initial results of this study, then perhaps long-term care facilities could implement the use of VR for their older patrons.

### Limitations

Although some of the enumerated findings are promising, this study had several limitations. First, many of the quantitative and qualitative measures were subjective and completed by the participants. Given that the participants completed fewer post-VR questionnaires than corresponding activities, the complete data set could not be analyzed after every activity. In addition, although the VR BA participants’ aforementioned feasibility data were collected from the headset, the participants in the traditional BA arm self-reported their real-life activities, which always yields a potential for inaccuracy. Similarly, although the PHQ-9 is a self-report measure, because of the remote nature of the study, the protocol director shared her screen with the VR BA and traditional BA participants and read the questions aloud to the TAU control participants over the phone while all participants verbally answered the 9 questions. This method may have resulted in less accurate reporting if the participants felt inclined to respond in a certain way. In addition, as there were no follow-ups, it is unknown whether the mood gains that the participants reported were lasting.

Second, one of the largest obstacles to the study design was recruitment. Although the goal was to randomize 30 participants into one of the 3 study arms, only 13 were randomized because other potential participants were excluded based on ineligibility, declining to participate, or being lost to follow-up. This difficulty in recruitment may be due in part to the COVID-19 pandemic and subsequent telehealth design, with people not wanting to participate in an unpaid study during this transition. It may also be due to lack of funding and an inability to broadly advertise but could be an inherent problem with depression studies where comorbidities and misdiagnoses are common and cause exclusion from controlled studies. Given the difficulties in recruiting enough participants to conduct a powered RCT and the subsequent small sample size, the results may not be generalizable and do not indicate causality. The results may also not be applicable to all populations struggling with symptoms of depression owing to the heterogeneity of the disorder.

### Conclusions

This was the first study of its kind, a historical first step in applying VR to a clinical population with MDD. Although technology is becoming increasingly popular and many studies have been conducted to analyze the feasibility and efficacy of using VR as an adjunct to or method of delivering mental health interventions for a variety of mental health disorders, this is the first study to analyze the feasibility and initial clinical efficacy of using VR as a method of engaging in pleasurable or mastery activities in conjunction with a brief BA protocol for individuals diagnosed with MDD.

This study illustrated that using VR as a method of administering BA in conjunction with a brief BA protocol for individuals diagnosed with MDD was feasible and that this intervention was able to integrate seamlessly into a telehealth design during a global pandemic. This study also illustrated that using VR as a method of administering BA in conjunction with a brief BA protocol was acceptable and tolerable for participants diagnosed with MDD.

The findings of this study demonstrate that clinicians can offer VR BA as a way for patients to experience pleasurable activities in conjunction with BA treatment to eliminate barriers that some patients may face when attempting traditional BA. VR may also be a viable alternative to psychiatric medications for some individuals given its high tolerability. In addition, given that many people do not receive adequate mental health care, VR could be a first step to help people improve their mood and increase activation while waiting to connect with a health care professional. VR BA may also be a way to operationalize and standardize BA and make it more acceptable for providers to deliver and improve the efficiency of practice. Implementation science examining VR BA is recommended.

## Appendix

Multimedia Appendix 1 CONSORT-eHEALTH checklist (V 1.6.1).

## Abbreviations

BA: behavioral activation
CONSORT: Consolidated Standards of Reporting Trials
EBP: evidence-based psychotherapy
HMD: head-mounted display
ITT: intention-to-treat
MDD: major depressive disorder
PHQ-9: Patient Health Questionnaire–9
RCT: randomized controlled trial
TAU: treatment as usual
VR: virtual reality
